# The importance of bone marrow biopsy for chronic myeloid leukemia classification—a case report

**DOI:** 10.1007/s12308-026-00684-8

**Published:** 2026-02-26

**Authors:** Natália Vital Gonçalves, Herton Luiz Alves Sales Filho, João Paulo Cabral de Magalhães Gomes, Guilherme Brasil Duffles Amarante, Gislaine Borba de Oliveira, Irene Lorand-Metze, Kátia Borgia Barbosa Pagnano, Guilherme Rossi Assis-Mendonça

**Affiliations:** 1 Division of Anatomic Pathology, Department of Pathology, School of Medical Sciences, University of Campinas (Unicamp), Campinas, Brazil; 2 Centro de Hematologia e Hemoterapia (Hemocentro-UNICAMP), Universidade Estadual de Campinas (UNICAMP), Campinas, Brazil; 3 Young Physician Leaders Program, National Academy of Medicine, Rio de Janeiro, Brazil

**Keywords:** Chronic myeloid leukemia, Lymphoid blast crisis, Biopsy, Histology, Bone marrow

## Abstract

Chronic myeloid leukemia (CML) is a myeloproliferative neoplasm characterized by the *BCR::ABL1* rearrangement, usually diagnosed by data of peripheral blood, bone marrow cytology, cytogenetics, and the detection of the *BCR:ABL1* rearrangement. An accurate diagnosis, including the precise phase of the disease, is essential to guide appropriate treatment. We present the case of a patient admitted at our Institution with the hypothesis of CML in chronic phase, based on clinical and bone marrow cytological findings. Bone marrow biopsy showed some areas with morphologic features of CML in chronic phase but also sheets of B lymphoblasts, compatible with lymphoid blast crisis. Flow cytometry detected 1.18% of lymphoblasts. This report clearly illustrates the importance of the examination of bone marrow by several techniques. It highlights the importance of the biopsy in recognizing anomalous immunomorphological patterns, which can lead to diagnostic and therapeutic changes in CML.

## Introduction

Chronic myeloid leukemia (CML) is a chronic myeloproliferative neoplasm (MPN) characterized by a translocation between chromosomes 9 and 22, t(9,22)(q34.1;q11.2). This results in the Philadelphia chromosome and *BCR::ABL1* fusion gene, with constitutively active tyrosine kinase activity [1, 2], which is the target for tyrosine kinase inhibitors. CML represents around 15% to 20% of leukemias in adults and can occur in any age group [3]. Usually it is diagnosed in chronic phase (more than 90% of cases), but some cases have a late diagnosis, either in accelerated phase (around 4 to 5% of cases) or in blast phase (BP). Of note, the latest World Health Organization (WHO 2022) recommends an emphasis on 2 forms of presentation of the disease (chronic phase and BP) omitting the accelerated phase from the classification [2].

Progression to BP from the chronic phase may occur both in untreated disease and as a result of progression or therapeutic resistance. BP presents mainly as myeloid (75%) phenotype, but in 25% of the cases, it has a lymphoid phenotype of B lineage. A bone marrow (BM) study (cytology and histology) is necessary in addition to cytogenetics and/or polymerase chain reverse transcription reaction to detect *BCR::ABL1* mRNA. Treatment at this stage consists of tyrosine kinase inhibitors (TKI), often associated with intensive chemotherapy protocols and allogeneic hematopoietic stem cell transplantation [4].

Here, we present the report of a patient admitted with CML apparently in chronic phase, but the BM biopsy disclosed large sheets of lymphoid blasts, characterizing the BP at initial diagnosis which could not be detected by BM cytology. Interestingly, both components (BM tissue in chronic phase and blastic phase) were seen in the same sample.

## Description of the case

**Fig. 1:**
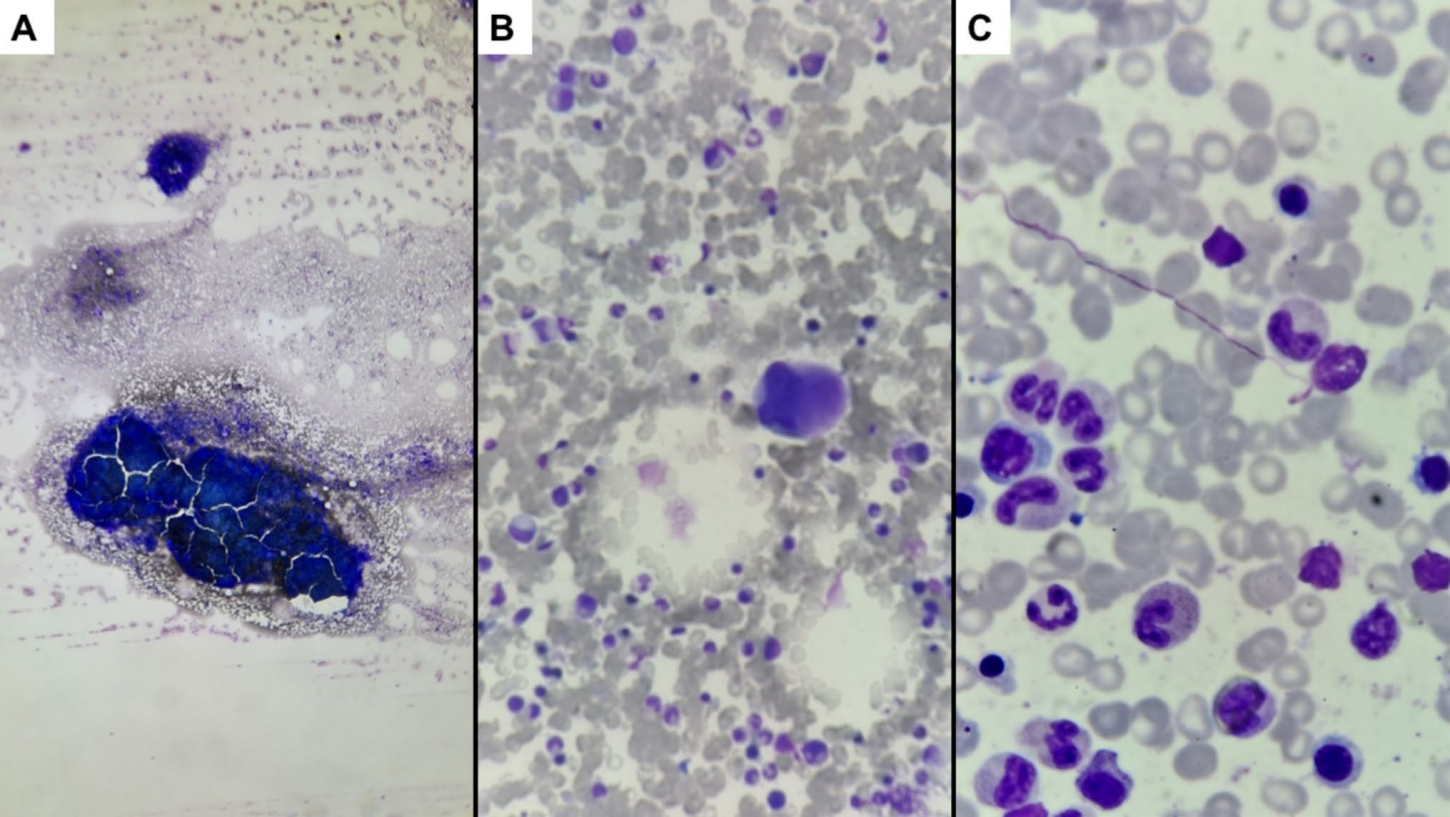
Representative photomicrographs of the bone marrow smear at the diagnosis. There was a global hypercellularity (**A** × 40 magnification) with some megakaryocytes (**B** × 200 magnification) and a predominance of mature granulocytes (**C** × 1000 magnification)

A 37-year-old woman sought hospital care in September 2024 due to bruises and asthenia that had lasted 1 month. The condition progressed after 3 weeks to ventilatory-dependent pain in the right hemithorax. On physical examination, the patient was pale (+/4 +), with pain in palpation of the last lower ribs on the right, without other findings. The spleen was palpable at the left costal board (15.6 cm by abdominal CT scan). Peripheral blood counts in the month of symptom onset showed a hemoglobin of 12.10 g/dL and total leukocyte count of 181.97 10 × 19^9^/l (6% blasts, 4% promyelocytes, 10% myelocytes, 10% metamyelocytes, 6% rods, 50% of neutrophils, 4% eosinophils, and 1% basophils). The platelet count was of 209.0 10 × 19^9^/l. Given the hypothesis of CML, the patient was admitted for investigation. The bone marrow smear showed a hypercellularity of mature granulocytes, with a G:E ratio of 5.43. No basophils were detected, and 3% of blasts were counted. Findings were suggestive of chronic phase CML (Fig. 1).

Cytogenetic analysis evidenced a 46,XX karyotype with t(9;22)(q34;q11) in 100% of 20 metaphases analyzed. Qualitative RT-PCR showed *BCR::ABL1* b2a2 rearrangement. So, the diagnosis of CML was established. Prognostic risk stratification was formally assessed using established scoring systems. The Sokal score was 0.9, corresponding to intermediate risk, whereas the ELTS score classified the patient as low risk.

**Fig. 2:**
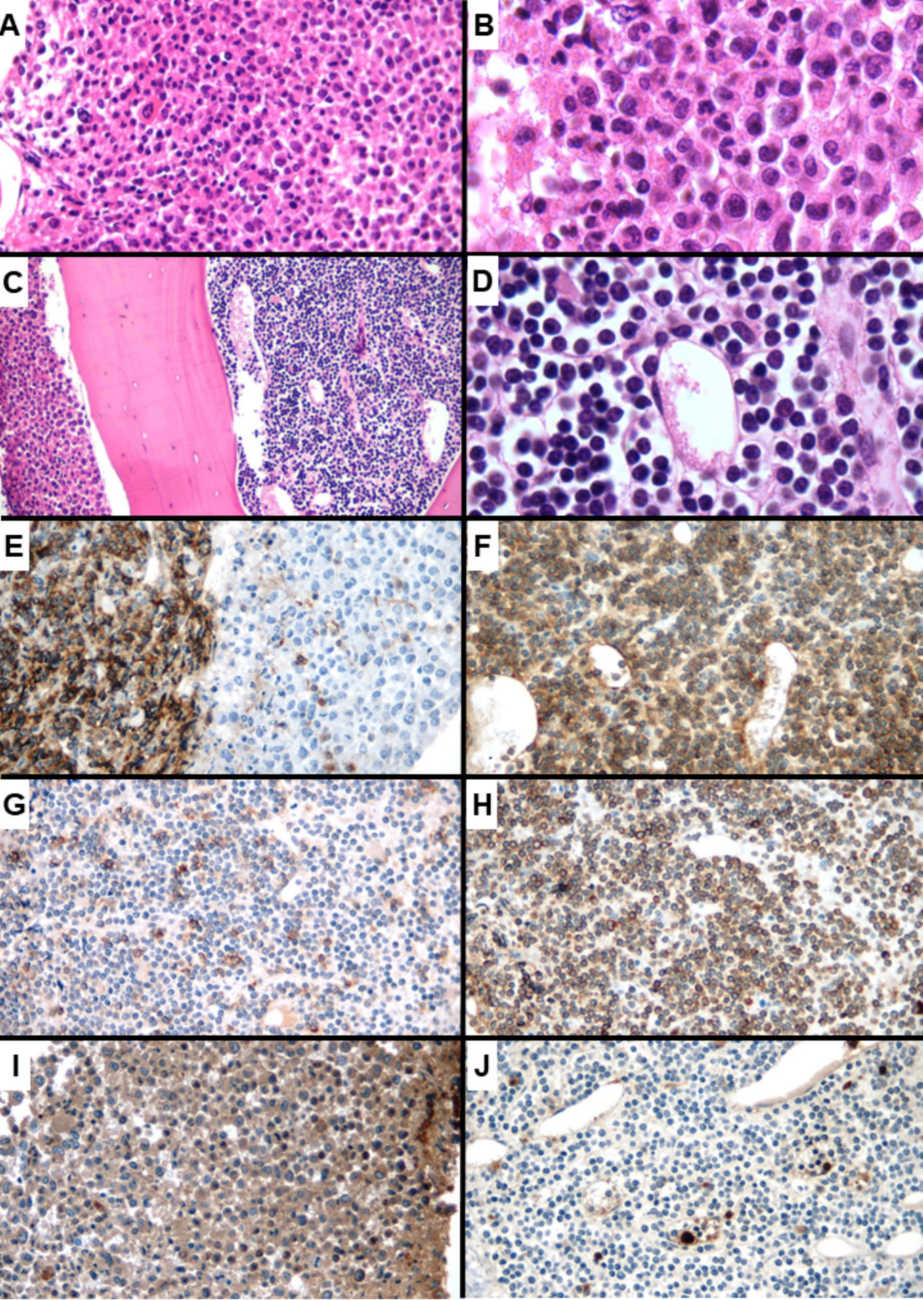
Bone marrow biopsy findings. Intense myeloid hypercellularity, associated with some hypo-lobulated megakaryocytes, consistent with CML (**A** × 400 magnification and **B** × 1000 magnification). An abrupt transition with sheets of lymphoblasts was seen (**C** × 200 magnification), evidenced by the presence of medium-sized monotonous round cells with scant cytoplasm (**D** × 1000 magnification). The immunohistochemical study showed diffuse staining for CD10 in the lymphoblasts on the left, contrasting with dim positivity on some mature granulocytes on the right (**E** × 400 magnification). The blasts showed strong positivity for CD79A (**F** × 400 magnification), partial reactivity for CD20 (**G** × 400 magnification), and strong staining for CD34 (**H** × 400 magnification). The staining for myeloperoxidase was positive in the areas compatible with chronic phase (**I** × 400 magnification) and negative on the lymphoblasts (**J** × 400 magnification)

The bone marrow biopsy showed an intense hypercellularity (> 95%), with a disrupted and heterogeneous architecture. In some intertrabecular spaces, an intense proliferation of myeloid elements was observed, with maturation arrest. There were also frequent megakaryocytes with a predominance of small, hypolobulated elements. However, in other intertrabecular spaces, a diffuse proliferation of small- to medium-sized elements with scant cytoplasm and round nuclei, suggestive of a lymphoid lineage, was seen (Fig. 2). Reticulin network was moderately increased (MF-2). The immunohistochemical study showed diffuse expression of myeloperoxidase in the proliferated myeloid elements. The small round cell component diffusely expressed CD79a, PAX-5, CD10 and CD34, and focally CD20 (Fig. 2). The final diagnosis from the bone marrow biopsy was of a CML in the chronic phase (70% of the sample), coexisting with a B-lymphoid BP in 30% of the sample.

**Fig. 3:**
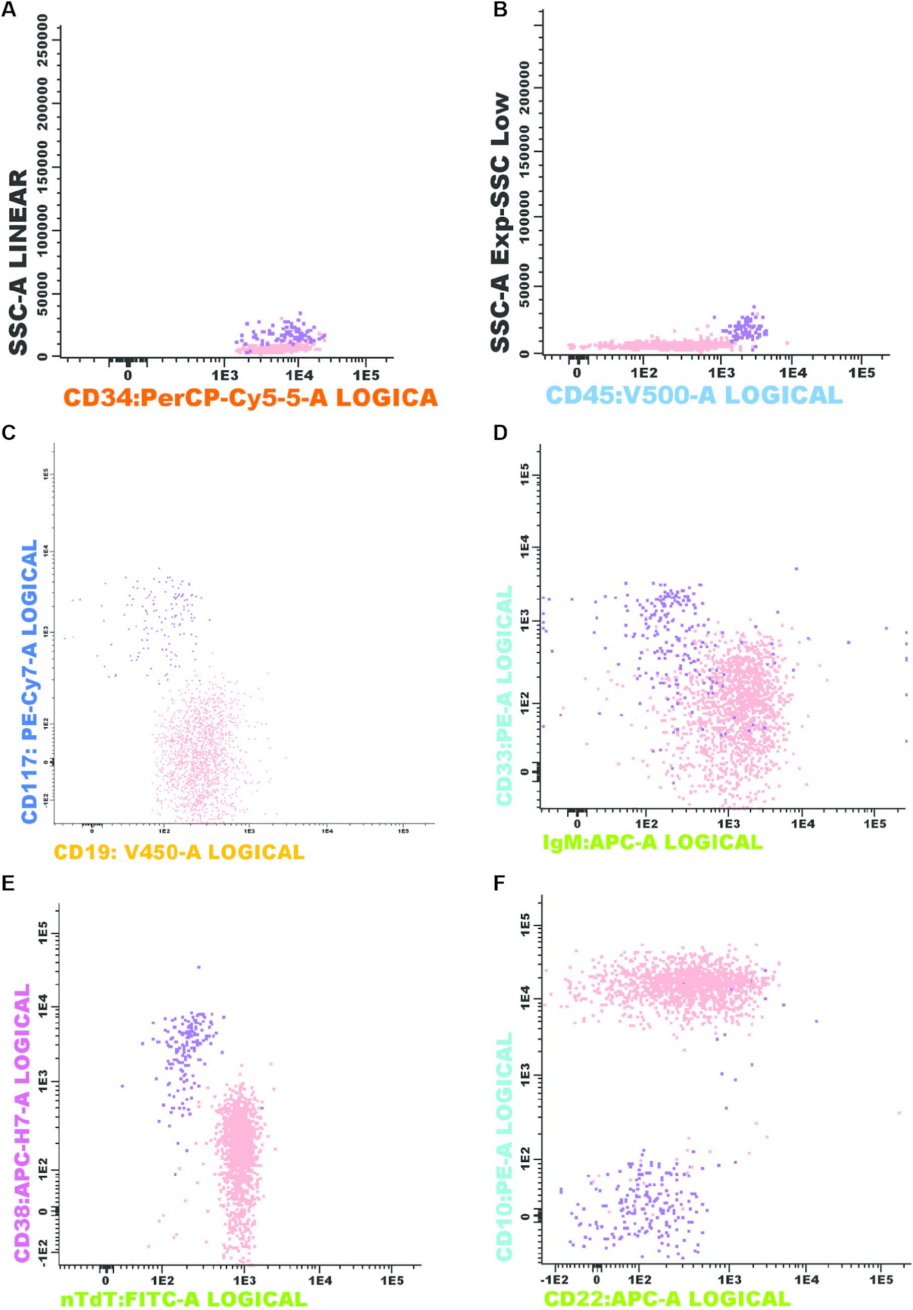
Immunophenotyping of the bone marrow by flow cytometry: two populations could be shown among the CD34 + cells (purple = myeloid; and salmon = lymphoid) (**A**). The myeloid blasts were positive for CD45 (**B**), CD117 (**C**), CD33 (**D**), and CD38 (**E**) and negative for lymphoid markers. The lymphoid blasts were negative for CD45 (**B**), CD38 (**E**), and myeloid markers and positive for CD19 (**C**), sIgM (**D**), nTdT (**E**), and CD22 and CD10 (**F**)

Flow cytometry is not routinely performed in our center for CML diagnosis but was done after the BM biopsy findings (Fig. 3). We could clearly distinguish two types of CD34 + cells: one myeloid (0.12%) and the other lymphoid (1.18%). This last one was clearly abnormal, as it was CD45-, SSC^low^, CD34^+^, CD19^+^, CD38-, CD22^partial^, CD10^+^, CD33-, and IgM^+^.

All these findings confirmed the diagnosis of CML in lymphoid blast crisis. The patient was treated with three cycles of chemotherapy (GRAAPH protocol with imatinib), followed by allogeneic hematopoietic stem cell transplant. Unfortunately, she developed a nosocomial infection and died of refractory septic shock after 20 days of the transplant.

## Discussion

According to the 2022 WHO and International Clinical Consensus (ICC) classifications, the diagnosis of BP in CML occurs when there are more than 20% blasts in peripheral blood and/or bone marrow cytology or extramedullary tumors with blast proliferation. However, there is a discrepancy among the classifications regarding the number of detectable lymphoblasts. In the ICC, the criterion for lymphoid BP is the presence of > 5% of morphologically apparent lymphoblasts, while in the WHO classification, it is characterized by the presence of increased lymphoblasts in peripheral blood or bone marrow, with no optimal cutoff [2, 5, 6].

Although the clinical relevance of very low percentages of lymphoblasts remains a matter of debate, accumulating evidence indicates that even minimal populations of aberrant lymphoblasts detected by flow cytometry may carry significant prognostic implications [7]. In a large cohort of newly diagnosed patients with chronic-phase chronic myeloid leukemia, Jiang et al. demonstrated that a flow cytometry–based aberrant lymphoblast (ALB) cutoff of 0.4%, determined by X-tile analysis, effectively stratified patients according to the risk of progression to lymphoid blast phase. Patients with ALB ≥ 0.4% exhibited a markedly increased cumulative incidence of lymphoid transformation, whereas those without detectable ALB showed excellent transformation-free survival. Notably, lymphoid transformation occurred early during tyrosine kinase inhibitor (TKI) therapy and was not observed below this threshold [7].

In the present case, only the biopsy, but not the smear, could confirm the presence of BP. The microscopic findings suggestive of blast crisis of a lymphoid lineage included the presence of large sheets of mononuclear cells, which could correspond to the accumulation of immature cells in extramedullary sites. Confirmation was made through an immunohistochemical study, with the expression of specific markers of lymphoblasts.

It is known that some patients with CML in chronic phase will progress to a BP. This may be due either to tumor genomic changes or to patients’ pharmacogenetic profiles of resistance to therapy [8]. The presence of BP at diagnosis is particularly uncommon (less than 5% of cases), with a higher prevalence (2–3 times) of myeloid BP compared to lymphoid [9, 10]. From a pathophysiological point of view, evolution to lymphoid BP does not appear to be associated with additional copies of the Ph chromosome or trisomy 8, but rather with specific additional mutations in genes such as *IKZF1*, *CDKN2A/B*, and *BCORL* [8].

The diagnosis of lymphoid BP, albeit uncommon, can be relatively straightforward if there is a clear lymphoblastic component evident in the bone marrow aspirate [11]. It is also possible the finding of different disease stages between the medullary study (showing chronic phase) and extramedullary tissue (showing lymphoid BP) [12, 13]. However, to the best of our knowledge, the simultaneous detection (“collision”) of the two components (chronic phase and lymphoid BP) in the same bone marrow biopsy sample of CML is very rare. Furthermore, the role that the biopsy played in the present case is noteworthy, since the initial percentage of blasts detected on BM smear and flow cytometric analysis was low. In this setting, a retrospective study of 508 patients with CML demonstrated that bone marrow biopsy was essential or helpful for diagnostic evaluation in 25% of cases. The need for biopsy was indeed more common in patients with BP, compared to the chronic phase of the disease, and in cases where aspirates were insufficient [14]. A possible explanation for the discrepancy in findings between our biopsy and bone marrow aspirate includes the presence of reticulogenesis. Also, the biopsy enabled examination of an undiluted, much larger sample of hemopoietic tissue that could disclose localized sheets of lymphoid blasts which comprised one-third of the area of the section. So, the distribution of the blasts was not homogeneous.

The immunohistochemical study in lymphoid BP of CML usually shows positivity for TdT and CD34 in lymphoid precursors, in addition to pan-B markers such as PAX-5 and CD79a in the case of “B” lymphoid crises [2]. Sometimes, a differential diagnosis between CML in lymphoid BP and Ph-positive acute lymphoid leukemia (ALL) is warranted, which is challenging and must take into account a combination of clinical, morphological, and molecular findings. In this sense, in the former, evidence is needed to support CML (presence of leukocytosis with maturation arrest of the granulocytic series, small and hypolobulated megakaryocytes). In contrast, the presence of small breakpoint mutations in *BCR* genes, patients of a younger age group, and no previous history of CML favor ALL [15].

The therapeutic strategy in CML consists of TKI in the chronic phase. As for patients in the BP who have not been previously treated, a complete hematological response with TKI occurs in only 50% of cases, and the addition of other drugs such as prednisolone and vincristine is often necessary in lymphoid BP. Despite this, responses are short, and relapses may occur in these patients after a few months, making it necessary to schedule an allogeneic bone marrow transplant if the patient is eligible [4]. In our case, the bone marrow biopsy was crucial for an accurate diagnosis, as it made it possible to include the patient in a more intensive treatment group, despite the final dismal outcome.

## Conclusion

Although B lymphoid BP is well characterized as a possible evolution of CML, the simultaneous observation, in the same biopsy, of morphological elements of the chronic phase and BP is exceptional. In addition, this case report highlights the importance of an integrated approach for the correct characterization of the disease phase and of the bone marrow biopsy for the CML phase classification. The immunohistochemical characterization and flow cytometry were essential to demonstrate the lineage of the immature cells. Finally, we emphasize the importance of constant contact between the hematopathologist and the clinical hematologist to identify concordant and discordant aspects between different samples and provide an integrated diagnosis.

## Data Availability

No datasets were generated or analysed during the current study.
